# Spatial–temporal trend for mother-to-child transmission of HIV up to infancy and during pre-Option B+ in western Kenya, 2007–13

**DOI:** 10.7717/peerj.4427

**Published:** 2018-03-13

**Authors:** Anthony Waruru, Thomas N.O. Achia, Hellen Muttai, Lucy Ng’ang’a, Emily Zielinski-Gutierrez, Boniface Ochanda, Abraham Katana, Peter W. Young, James L. Tobias, Peter Juma, Kevin M. De Cock, Thorkild Tylleskär

**Affiliations:** 1 Centre for International Health, University of Bergen, Bergen, Norway; 2 Division of Global HIV & TB (DGHT), U.S. Centers for Disease Control and Prevention, Nairobi, Kenya; 3 Division of Global HIV & TB (DGHT), U.S. Centers for Disease Control and Prevention (CDC), Atlanta, GA, USA; 4 University of Nairobi, Nairobi, Kenya

**Keywords:** Mother-to-child transmission, Pediatrics, Early infant diagnosis, Option B+, Spatial–temporal analysis, Geographical disparities

## Abstract

**Introduction:**

Using spatial–temporal analyses to understand coverage and trends in elimination of mother-to-child transmission of HIV (e-MTCT) efforts may be helpful in ensuring timely services are delivered to the right place. We present spatial–temporal analysis of seven years of HIV early infant diagnosis (EID) data collected from 12 districts in western Kenya from January 2007 to November 2013, during pre-Option B+ use.

**Methods:**

We included in the analysis infants up to one year old. We performed trend analysis using extended Cochran–Mantel–Haenszel stratified test and logistic regression models to examine trends and associations of infant HIV status at first diagnosis with: early diagnosis (<8 weeks after birth), age at specimen collection, infant ever having breastfed, use of single dose nevirapine, and maternal antiretroviral therapy status. We examined these covariates and fitted spatial and spatial–temporal semiparametric Poisson regression models to explain HIV-infection rates using R-integrated nested Laplace approximation package. We calculated new infections per 100,000 live births and used Quantum GIS to map fitted MTCT estimates for each district in Nyanza region.

**Results:**

Median age was two months, interquartile range 1.5–5.8 months. Unadjusted pooled positive rate was 11.8% in the seven-years period and declined from 19.7% in 2007 to 7.0% in 2013, *p* < 0.01. Uptake of testing ≤8 weeks after birth was under 50% in 2007 and increased to 64.1% by 2013, *p* < 0.01. By 2013, the overall standardized MTCT rate was 447 infections per 100,000 live births. Based on Bayesian deviance information criterion comparisons, the spatial–temporal model with maternal and infant covariates was best in explaining geographical variation in MTCT.

**Discussion:**

Improved EID uptake and reduced MTCT rates are indicators of progress towards e-MTCT. Cojoined analysis of time and covariates in a spatial context provides a robust approach for explaining differences in programmatic impact over time.

**Conclusion:**

During this pre-Option B+ period, the prevention of mother to child transmission program in this region has not achieved e-MTCT target of ≤50 infections per 100,000 live births. Geographical disparities in program achievements may signify gaps in spatial distribution of e-MTCT efforts and could indicate areas needing further resources and interventions.

## Introduction

An estimated 2.6 million children were living with HIV in 2014, making mother to child transmission of HIV (MTCT) an important contributor to the overall global burden of HIV (Joint United Nations Programme on HIV/AIDS (UNAIDS), 2015b). Between 2000 and 2014, new pediatric infections declined by up to 50% amidst some geographical variations (Joint United Nations Programme on HIV/AIDS (UNAIDS), 2016b). Worldwide, 220,000 children became newly infected with HIV in 2014, the vast majority (190,000) of whom were living in sub-Saharan Africa (SSA) (Joint United Nations Programme on HIV/AIDS (UNAIDS), 2015a). Kenya was estimated to have 101,000 children living within HIV in 2012 (Joint United Nations Programme on HIV/AIDS (UNAIDS), 2012), with 13,000 new infections annually (National AIDS and STI Control Programme (NASCOP), 2014), and has the fifth highest HIV-incidence among children in SSA (Joint United Nations Programme on HIV/AIDS (UNAIDS), 2014a). Effective implementation of prevention of mother to child transmission of HIV (PMTCT) programs are therefore critical to reduced HIV transmission and elimination of mother-to-child transmission of HIV (e-MTCT).

Great strides have been made in e-MTCT, for example, new HIV infections have been reduced by nearly half among children in the 21 priority countries with the highest HIV-burden in SSA (Joint United Nations Programme on HIV/AIDS (UNAIDS), 2015c). This has been realized by implementing the United Nations four-pronged strategy for PMTCT: preventing new HIV infections among women of childbearing age; preventing unintended pregnancies among women living with HIV; preventing HIV transmission from a woman living with HIV to her baby; and providing appropriate treatment, care and support to mothers living with HIV, their children and families (Joint United Nations Programme on HIV/AIDS (UNAIDS), 2015b). The same four-pronged strategy is adopted in the Kenya PMTCT guidelines (National AIDS and STI Control Programme (NASCOP), 2012). In Kenya, the burden of HIV among pregnant women is high. In 2013 alone, Kenya was ranked sixth among 21 countries in terms of HIV-positive women delivering in health facilities with an estimated 79,000 HIV-positive women giving birth (or pregnant) (Joint United Nations Programme on HIV/AIDS (UNAIDS), 2015d). More recent estimate indicates that 79,500 (95% CI [70,100–91,200]) women are in need of PMTCT and overall MTCT rate as 8.3% (National AIDS and STI Control Programme Ministry of Health, National AIDS Control Council (NACC), 2015). It is therefore critical to prevent HIV transmission from women to infants and children.

In the period 2010–2016, Kenya has been ranked 10th in Eastern and southern Africa in progress towards reduction of HIV incidence among 0–14 year olds (Joint United Nations Programme on HIV/AIDS (UNAIDS), 2017). Between 2010 and 2015, final MTCT rate reduced by half from 17% in 2010 to 8% in National AIDS and STI Control Programme Ministry of Health, National AIDS Control Council (NACC) (2015). The UNAIDS 2016–2021 second e-MTCT strategy outlines the objectives to work towards zero new HIV infections among children, and improved mother survival by 2020 (Joint United Nations Programme on HIV/AIDS (UNAIDS), 2015e). The impact target for e-MTCT has been set as ≤50 new pediatric HIV infections per 100,000 live births and a transmission rate of <5% in breastfeeding populations and <2% in nonbreastfeeding populations (World Health Organization, 2014). However, this transmission rate should be calculated as “final” infection status in breastfeeding populations. Measuring MTCT rates is therefore an essential indicator of PMTCT program success.

The UNAIDS fast-track 90–90–90 strategy (Joint United Nations Programme on HIV/AIDS (UNAIDS), 2014b) requires a location–population approach so as to refocus efforts in containing the HIV epidemic (Joint United Nations Programme on HIV/AIDS (UNAIDS), 2015a), hence the emphasis on “where,” to identify pockets needing focused interventions. Program data are often reported at country-level and rarely in more refined subnational geographical areas. Ignoring the influence of interactions across neighboring subnational units such as districts and excluding temporal variables and covariates in analyses may not sufficiently explain access and coverage. Taking these considerations into account is important to improve assessment of gains towards e-MTCT through measuring MTCT and early infant diagnosis (EID) coverage over space, time and at a more granular level.

In Kenya, the EID program has expanded since initiation in 2004 and has been accompanied by accreditation of seven laboratories nationally with capacity to conduct polymerase chain reaction (PCR) testing of HIV. The expansion of EID was also commensurate with the recommendation for use of lifelong ART (Option B+) for all pregnant and breastfeeding women in Kenya (National AIDS and STI Control Programme (NASCOP), 2012), which fully came into effect in 2014. Although dried-blood-spot (DBS) PCR testing was available since 2005, there are minimal data available about the program scale-up, and the characteristics of children tested and identified as HIV-infected prior to 2007. The national EID database is useful for decision making at the national level but documenting regional variations has not been feasible with limited availability of programmatic, spatial, and spatial–temporal data. Various tools have been developed and applied in spatial–temporal analysis of diseases (Rushton, 2003; Auchincloss et al., 2012). In this analysis, we have used spatial–temporal analysis methods to present seven years of EID data collected from pre-Option B+ period of January 2007–November 2013 and to demonstrate usefulness of spatial–temporal trend analysis in identifying areas that may need further programmatic efforts.

## Methods

### Study area

Nyanza region in western Kenya is approximately 2,549 km^2^ with a population density of 440/km^2^ (Kenya National Bureau of Statistics (KNBS), 2010b), and the highest adult HIV prevalence in Kenya (National AIDS and STI Control Programme (NASCOP), 2014). In 2004, when the President’s Emergency Plan for AIDS Relief started in Kenya, and prior to 2007, the region was divided into 12 districts to facilitate geographical PMTCT programmatic planning. The 12 districts were: Bondo, Kisii, Gucha, Homa Bay, Kisumu, Kuria, Migori, Nyamira, Nyando, Rachuonyo, Siaya, and Suba. We have aggregated data at district level from 924 facilities from where EID samples were collected. These data represented nearly 90% (924/1,072) of the health facilities implementing the PMTCT program in Nyanza region by 2013.

### Population and live births estimates

Parents or guardians of infants known or suspected of being perinatally HIV-exposed infants were asked for consent to diagnostic virologic testing of their children as part of routine HIV care. The population of infants and children tested included those whose mothers were diagnosed with HIV infection before or during pregnancy, at delivery, and up to the time the mothers brought their children for the first HIV test (usually at six weeks for first routine vaccinations). We estimated the number of live births based on the 2009 census (Kenya National Bureau of Statistics (KNBS), 2010a), using an estimated annual growth rate of 4.1%, 2012–2030 (United Nations Children’s Fund (UNICEF), 2016), which gives a crude birth rate of 41 births per 1,000 population. We used this projection to validate the number of women tested as proxy for pregnant women presenting in the clinics in 2013 as basis for the calculation of standardized MTCT rates per 100,000 live births.

### EID pilot program procedures in Nyanza

Use of the EID patient data collection tool was first implemented in health facilities requesting the Kenya Medical Research Institute (KEMRI) Kisumu laboratory to perform EID testing in 2006. As part of routine service delivery, this form was completed by clinicians at facilities requesting DBS PCR HIV testing and accompanied each specimen to the laboratory. Subsequently, the national EID form was developed by the National AIDS and STI Control Program and these forms were used by clinicians and accompanied specimens for HIV testing. EID results were added to the form once laboratory testing was completed. One copy of the form was sent back to the health facility for patient management and the second copy scanned into an electronic, password-protected database. The data presented in this analysis are for infants undergoing first EID PCR test.

### Laboratory procedures

Between January 2007 and November 2013, blood samples were collected from infants presenting at health facilities in Nyanza region as part of a study: “Evaluation of HIV EID testing in Kenya,” and transported to the Kisumu HIV laboratory for HIV diagnosis. Testing was done using PCR on either COBAS Ampliprep/COBAS TaqMan HIV-1 assay (TaqMan; Roche Diagnostics, Mannheim, Germany) or Abbot (Abbott RT; Abbott Diagnostics, Wiesbaden, Germany) platforms. These results were returned to the submitting facility for clinical action and notification of the parent/guardian.

### Measures

#### Mother to child transmission rates

The transmission rates calculated reflect MTCT up to infancy since we used the first PCR testing and included infants who were up to 12 months old at HIV diagnosis. To calculate MTCT rate, the main outcome variable, the number of infants with PCR-positive HIV-test results was taken as the numerator and divided by the total number of HEI tested during the study period to determine rates applicable within the geographic regions. Adjusted rates were further calculated using R version 3.2.3 (R Core Team, 2015) implemented in RStudio version 0.99.903 (RStudio, 2016). Standardized MTCT rates per 100,000 live births were calculated as: (absolute transmission (number infected)/women tested for HIV in 2013) × 100,000.

#### Covariates selection

The following infant and maternal factors were included in the spatial–temporal model as covariates: early diagnosis (<8 weeks after birth), age of the child at specimen collection, infant ever having breastfed, use of single dose nevirapine (sdNVP), and maternal antiretroviral treatment (ART) status. In the descriptive outputs and logistic models, maternal regimen was categorized as: (a) sdNVP, (b) ART for prophylaxis = AZT that started at 14 weeks, intrapartum sdNVP and first dose of AZT+3TC and during postpartum period, daily AZT+3TC for seven days. This is also referred to as *short course* when ARVs starting at 14 weeks gestation and continued through the intrapartum and childbirth if not breast feeding or until one week after cessation of all breastfeeding, and (c) ART for treatment = Triple ARVs for women who had CD4 cell counts of ≤350 cells/mm^3^ starting as soon as diagnosed and continued for life (National AIDS and STI Control Programme (NASCOP), 2012; World Health Organization (WHO), 2012). This was the precursor of Option B+ which did not start in Kenya until June 2014.

### Analytical approaches

#### Statistical analyses

To explore associations of maternal and infant related factors to HIV acquisition, we conducted bivariate and multivariable logistic regression analyses using Stata 14.2 (Stata Corporation, College Station, TX, USA). Variables that were significant in the bivariate model were included in the multivariable model. Extended Cochran–Mantel–Haenszel test of trend for proportions was used to assess trend for both outcome and explanatory variables. These analyses informed variables to include in the spatial and spatial–temporal analyses.

#### Spatial and spatial–temporal model fitting in R-INLA

We performed spatial and spatial–temporal analyses in R version 3.2.3 (R Core Team, 2015) implemented in RStudio© version 0.99.903 (RStudio, 2016) using integrated nested Laplace approximation (R-INLA) package (Blangiardo et al., 2013), to explore covariates to explain observed spatial–temporal trends using semiparametric Poisson regression.

We fitted five Poisson regression models as follows:

1. To assess general associations of covariates with the outcome variable, we fitted a nonspatial generalized linear model.

To assess spatial relationships, we fitted semiparametric Poisson regression models as follows:

- a spatial model without covariates,
- a spatial–temporal model without covariates,
- a spatial–nontemporal model with covariates,
- and finally, a spatial–temporal model with covariates according to Blangiardo, Cameletti, and Rue (Blangiardo et al., 2013). This final model allows for an interaction of space, time, and covariates, which would explain differences in the time trend of MTCT rates for districts in Nyanza region.

For each of these spatial models (2–5), we used Bayesian deviance information criterion (DIC) according to Spiegelhalter et al. (2002) and Spiegelhalter, Best & Carlin (1998) to evaluate the strength of the fits combined with examination of posteriors generated through plotting. Bayesian analytic approach is conditional on the appropriateness of an assumed probability model. Hence, DIC is a useful tool to satisfy that our assumptions are reasonable approximation to reality. For the best fitting model, we conducted sensitivity analyses to assess the robustness of the priors (assumed probability distribution) selected. Full derived models are presented in Appendix 1.

### Mapping

Each of the samples had at a minimum locator information which contained the name of the facility and district. Using spatial join technique, both the outcome and covariates data were aggregated at district (currently called subcounty) level to provide rates for spatial, and spatial–temporal analysis and mapping. For the final maps, we selected the best fitting model and extracted fitted estimates from R-INLA and mapped these rates as shaded choropleth maps to show the 12 districts by year of HIV-diagnosis (with color intensity depicting higher rates) using Quantum GIS version 2.14.1 (QGIS Development Team, 2016).

### Ethical clearance and informed consent

Ethical approval for the study was obtained from the KEMRI and the United States Centers for Disease Control and Prevention. Further consent was not necessary since these data were routinely collected deidentified data from routine clinic services.

## Results

### Trends in programmatic uptake and MTCT rates

**Figure 1:**
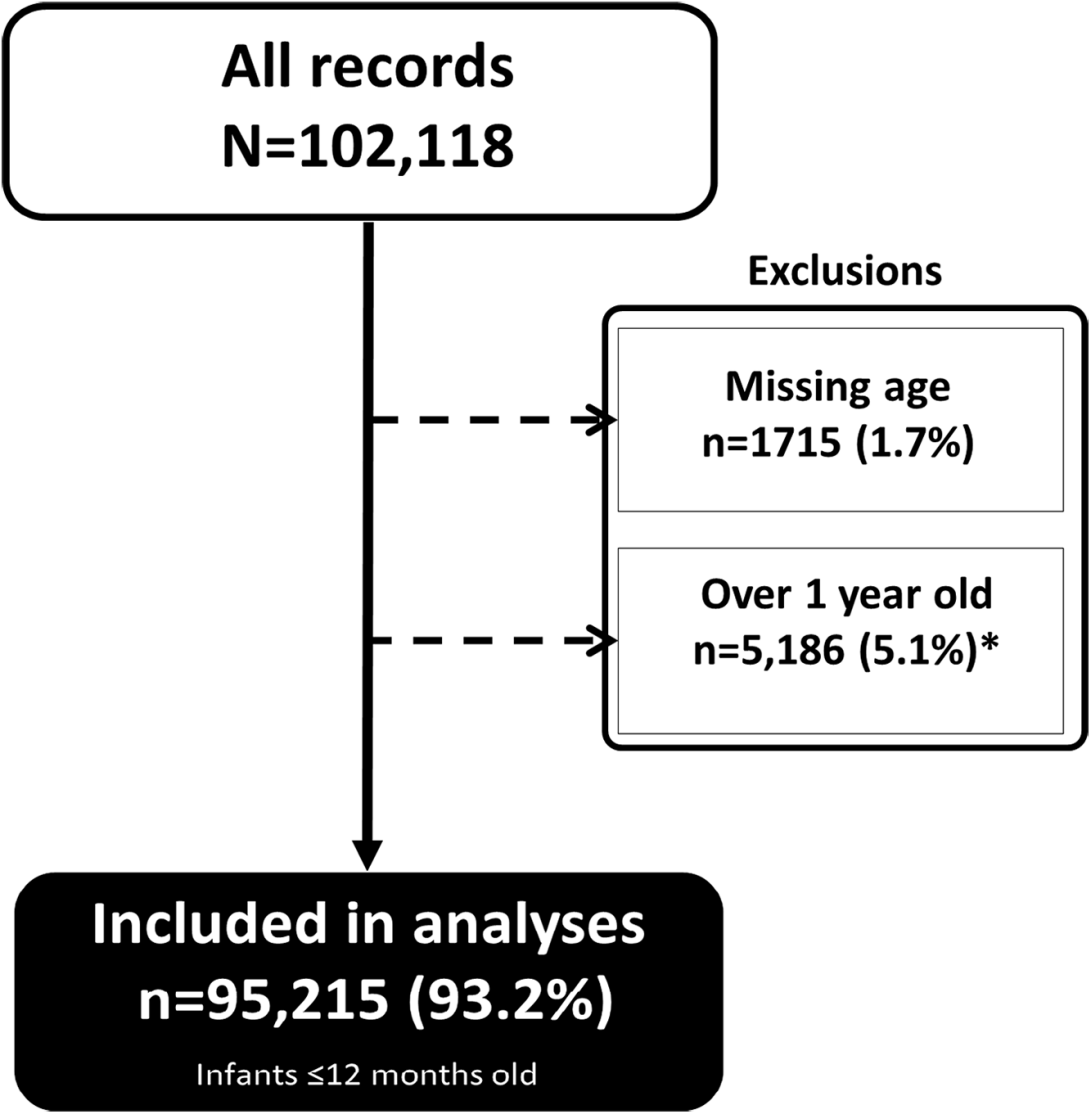
Infants included in the analyses. A total of 95,215 infants ∼93.2% of all infants and children at HIV diagnosis were included in the analyses.

These results represent data from 95,215 infants and equally distributed by sex. These were ∼93.2% of all infants and children at HIV diagnosis (Fig. 1).

Most of the infants were tested in 2011 and 2012. Median age at HIV testing was two months, interquartile range 1.5–5.8 months (Table 1). About three quarters (75.1%) of the infants were under six months old at the point of testing and the majority (60.0%) were tested at the maternity/postnatal ward. Median age at HIV testing decreased from approximately three months in 2007–2009 to under two months by 2013, *p* < 0.01.

**Table 1:** Characteristics of infants and mothers for HEI tested for HIV and trends in Western Kenya, 2007–2013.

Characteristics	Total	2007	2008	2009	2010	2011	2012	2013	*p*-Value
Total tested each year	95,215	5,090	6,628	10,437	13,236	21,981	20,714	17,129	–
Sex									0.249
Male	47,733 (50.1%)	2,507 (49.3%)	3,293 (49.7%)	5,138 (49.2%)	6,696 (50.6%)	11,201 (51%)	10,328 (49.9%)	8,570 (50%)	
Female	47,482 (49.9%)	2,583 (50.7%)	3,335 (50.3%)	5,299 (50.8%)	6,540 (49.4%)	10,780 (49%)	10,386 (50.1%)	8,559 (50%)	
Age (median, IQR)	2.0 (1.5, 5.8)	2.8 (1.8, 6.0)	3.0 (1.5, 6.0)	3.0 (1.5, 6.0)	2.0 (1.5, 6.0)	2.0 (1.5, 5.0)	1.8 (1.5, 4.0)	1.8 (1.5, 4.0)	<0.001
Age (months)									<0.001
Under six months	71,486 (75.1%)	3,677 (72.2%)	4,626 (69.8%)	6,946 (66.6%)	9,458 (71.5%)	16,800 (76.4%)	16,334 (78.9%)	13,645 (79.7%)	
6–12 months	23,729 (24.9%)	1,413 (27.8%)	2,002 (30.2%)	3,491 (33.4%)	3,778 (28.5%)	5,181 (23.6%)	4,380 (21.1%)	3,484 (20.3%)	
Entry point*									<0.001
OPD	1,089 (1.1%)	0 (0%)	0 (0%)	0 (0%)	0 (0%)	278 (1.3%)	454 (2.2%)	357 (2.1%)	
Pediatric ward	239 (0.3%)	0 (0%)	0 (0%)	0 (0%)	0 (0%)	98 (0.4%)	89 (0.4%)	52 (0.3%)	
Maternity	57,171 (60.0%)	2,285 (44.9%)	2,997 (45.2%)	5,269 (50.5%)	7,034 (53.1%)	12,727 (57.9%)	14,174 (68.4%)	12,685 (74.1%)	
HBTC	363 (0.4%)	1 (0%)	3 (0%)	15 (0.1%)	35 (0.3%)	264 (1.2%)	24 (0.1%)	21 (0.1%)	
DTC/PITC	841 (0.9%)	369 (7.2%)	265 (4%)	181 (1.7%)	19 (0.1%)	2 (0%)	4 (0%)	1 (0%)	
VCT (Site/Mobile)	123 (0.1%)	31 (0.6%)	63 (1%)	18 (0.2%)	6 (0%)	0 (0%)	3 (0%)	2 (0%)	
Other	3,975 (4.2%)	792 (15.6%)	732 (11%)	364 (3.5%)	689 (5.2%)	890 (4%)	284 (1.4%)	224 (1.3%)	
Unknown	6,331 (6.6%)	154 (3%)	330 (5%)	752 (7.2%)	1,078 (8.1%)	2,037 (9.3%)	1,265 (6.1%)	715 (4.2%)	
Infant breastfed									<0.001
Yes	75,643 (79.4%)	3,324 (65.3%)	4,568 (68.9%)	7,271 (69.7%)	9,952 (75.2%)	17,678 (80.4%)	17,785 (85.9%)	15,065 (88%)	
No	2,126 (2.2%)	973 (19.1%)	985 (14.9%)	140 (1.3%)	8 (0.1%)	19 (0.1%)	1 (0%)	0 (0%)	
Unknown	3,600 (3.8%)	729 (14.3%)	614 (9.3%)	631 (6%)	432 (3.3%)	732 (3.3%)	278 (1.3%)	184 (1.1%)	
Did mother receive ARV?									<0.001
Yes	45,865 (92.8%)	1,598 (52.8%)	3,128 (64.4%)	4,314 (94.6%)	5,362 (99.5%)	9,999 (99%)	11,084 (100%)	10,380 (100%)	
No	3,182 (6.4%)	1,279 (42.2%)	1,568 (32.3%)	222 (4.9%)	23 (0.4%)	90 (0.9%)	0 (0%)	0 (0%)	
Unknown	352 (0.7%)	152 (5%)	162 (3.3%)	26 (0.6%)	5 (0.1%)	7 (0.1%)	0 (0%)	0 (0%)	
Mother alive/dead^†^									<0.001
Alive	91,610 (96.2%)	4,361 (85.7%)	6,014 (90.7%)	9,806 (94%)	12,800 (96.7%)	21,248 (96.7%)	20,436 (98.7%)	16,945 (98.9%)	
Dead	5 (0%)	0 (0%)	0 (0%)	0 (0%)	4 (0%)	1 (0%)	0 (0%)	0 (0%)	
Unknown	3,600 (3.8%)	729 (14.3%)	614 (9.3%)	631 (6%)	432 (3.3%)	732 (3.3%)	278 (1.3%)	184 (1.1%)	
Maternal regimen^‡^									<0.001
sdNVP only	2,763 (6.7%)	0 (0%)	101 (16.7%)	498 (12.5%)	471 (8.8%)	680 (6.8%)	583 (5.3%)	430 (4.1%)	
ART for prophylaxis	16,185 (39.2%)	2 (66.7%)	191 (31.6%)	1,505 (37.8%)	1,611 (30.1%)	3,912 (39.4%)	4,558 (41.1%)	4,406 (42.5%)	
ART for treatment	22,389 (54.1%)	1 (33.3%)	313 (51.7%)	1,974 (49.7%)	3,272 (61.1%)	5,344 (53.8%)	5,941 (53.6%)	5,544 (53.4%)	
Testing facility									<0.001
Community/VCT	388 (0.4%)	0 (0%)	0 (0%)	12 (0.1%)	40 (0.3%)	291 (1.3%)	33 (0.2%)	12 (0.1%)	
Dispensaries/HC/Clinics/NH	53,390 (56.1%)	1,792 (35.2%)	2,523 (38.1%)	4,354 (41.7%)	6,849 (51.7%)	13,006 (59.2%)	13,474 (65%)	11,392 (66.5%)	
Subdistrict hospitals	9,255 (9.7%)	385 (7.6%)	704 (10.6%)	1,368 (13.1%)	1,372 (10.4%)	2,155 (9.8%)	1,831 (8.8%)	1,440 (8.4%)	
District hospitals	21,267 (22.3%)	1,939 (38.1%)	2,207 (33.3%)	3,188 (30.5%)	3,305 (25%)	4,503 (20.5%)	3,500 (16.9%)	2,625 (15.3%)	
Level 5 hospitals	5,313 (5.6%)	714 (14%)	697 (10.5%)	597 (5.7%)	804 (6.1%)	900 (4.1%)	826 (4%)	775 (4.5%)	
FBO	5,602 (5.9%)	260 (5.1%)	497 (7.5%)	918 (8.8%)	866 (6.5%)	1,126 (5.1%)	1,050 (5.1%)	885 (5.2%)	

**Notes:**

* Definitions: OPD, outpatient department; HBTC, home based testing and counseling; VCT, voluntary counseling and testing; SdNVP, single dose Nevirapine; ART, antiretroviral therapy; AZT, Zidovudine; 3TC, Lamivudine; HC, health center; NH, nursing home; FBO, faith-based organization.

† At time of sample collection.

‡ ART for prophylaxis.

**Figure 2:**
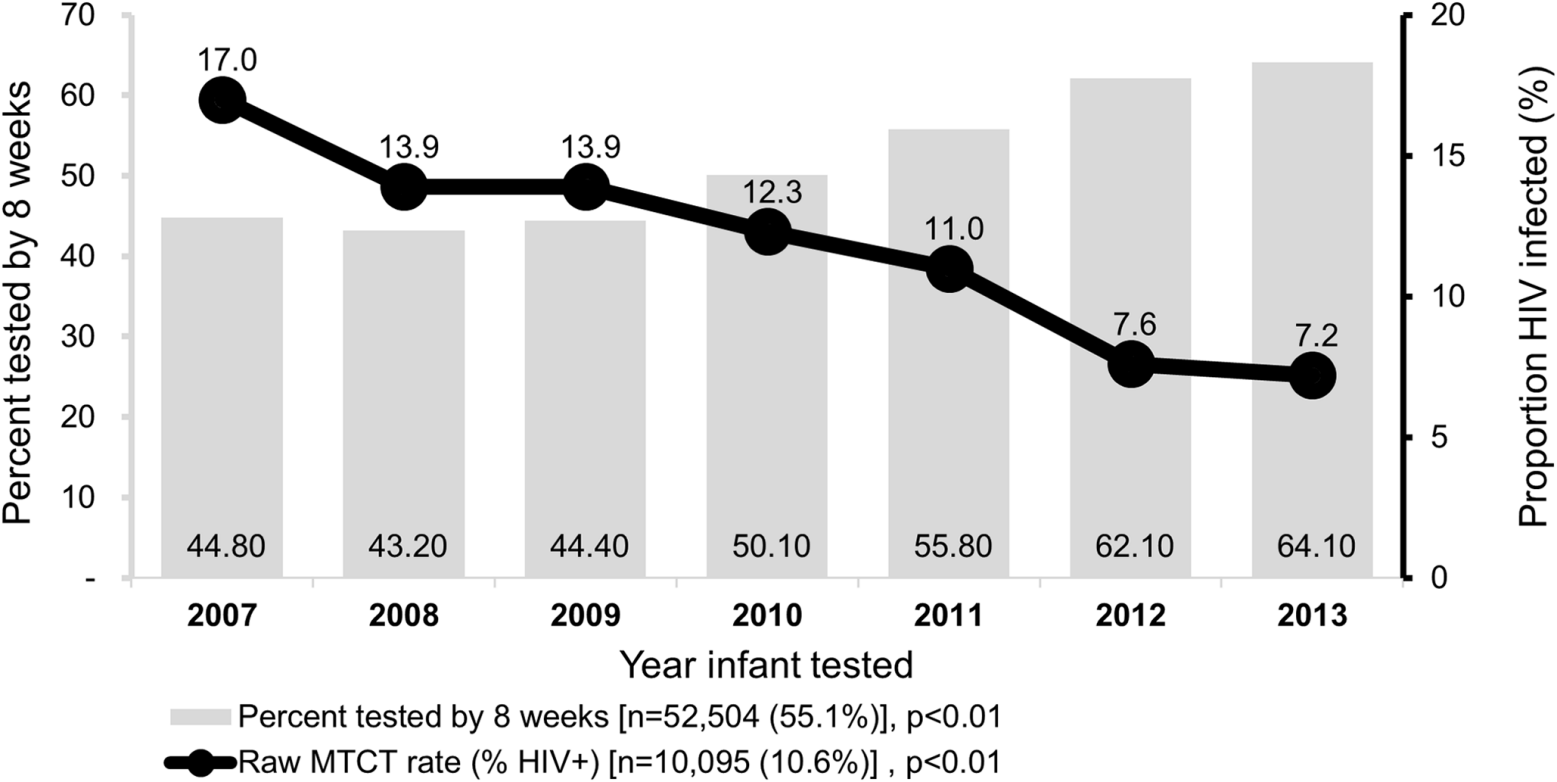
Trends in HIV diagnosis and raw MTCT among infants in Western Kenya, 2007–2013. Primary *y*-axis shows proportion of infants tested at <8 weeks after birth while secondary *y*-axis shows proportion of HIV-infected infants out the tested.

The proportion of infants tested at maternity or postnatal ward increased from 44.9% to 74.1% by year 2013, *p* < 0.01. The proportion of infants reported to have been breastfed increased over the years from 65.3% in 2007 to 88.0% in 2012, *p* < 0.01. The proportion of mothers receiving ART for treatment (during pregnancy or breastfeeding period) increased from 52.8% in 2007 to 100% in 2013, *p* < 0.001; the proportion of mothers alive at the time of infant testing over the same period increased from 85.7% in 2007 to 98.9% by 2013, *p* < 0.001. Use of ART for treatment increased over the seven-year period from to 61.1% by 2013, *p* < 0.001. Overall, early testing (at <8 weeks after birth) was 55.5% and increased from 44.8% in 2007 to 64.1% in 2013, *p* < 0.01 (Fig. 2).

The unadjusted HIV-infection rates decreased from 17.0% in 2007 to 7.2% in 2013, *p* < 0.01.

### Association of infants and maternal factors with HIV infection

In multivariable analysis; infants tested in 2009–2012 compared to those tested in 2013, late diagnosis (beyond eight weeks after birth), and use of sdNVP, ART for prophylaxis compared to ART for treatment were associated with MTCT (Table 2).

**Table 2:** Factors associated with mother to child transmission of HIV (MTCT) among infants in western Kenya, 2007–2013.

Characteristic	Total (*n*)	Positive, *n* (%)	Unadjusted		Adjusted	
			OR*	[95% CI]	aOR^†^	[95% CI]
Total	95,215	10,095				
Year						
2007	5,090	867 (17%)	2.7	(2.4, 2.9)	(Omitted)	(Omitted)
2008	6,628	923 (13.9%)	2.1	(1.9, 2.3)	1.8	(1.3, 2.4)
2009	10,437	1,454 (13.9%)	2.1	(1.9, 2.3)	1.5	(1.3, 1.8)
2010	13,236	1,632 (12.3%)	1.8	(1.7, 2.0)	1.6	(1.4, 1.8)
2011	21,981	2,414 (11%)	1.6	(1.5, 1.7)	1.4	(1.3, 1.6)
2012	20,714	1,574 (7.6%)	1.1	(1.0, 1.2)	1.0	(0.9, 1.1)
2013	17,129	1,231 (7.2%)	ref.^‡^		ref.	
Sex						
Male	47,733	4,811 (10.1%)	ref.		ref.	
Female	47,482	5,284 (11.1%)	1.1	(1.1, 1.2)	1.2	(1.1, 1.3)
Age (months)					^¶^	
Under six months	71,486	5,964 (8.3%)	ref.			
6–12 months	23,729	4,131 (17.4%)	2.3	(2.2, 2.4)		
Age at diagnosis						
Under/= eight weeks	52,504	3,307 (6.3%)	ref.		ref.	
Over eight weeks	42,711	6,788 (15.9%)	2.8	(2.7, 3.0)	2.5	(2.3, 2.7)
Maternal regimen						
sdNVP only	2,763	279 (10.1%)	2.0	(1.8, 2.3)	1.7	(1.5, 2)
ART for prophylaxis	11,634	1,199 (7.4%)	1.5	(1.3, 1.6)	1.5	(1.3, 1.6)
ART for treatment	4,551	1,171 (5.2%)	ref.		ref.	
Infant breastfed					^¶^	
Yes	75,643	7,703 (10.2%)	ref.			
No	2,126	327 (15.4%)	1.6	(1.4, 1.8)		
Unknown	17,446	2,065 (11.8%)	n.i^§^			
Mother received ARV					^¶^	
Yes	45,865	3,225 (7%)	ref.			
No	3,182	632 (19.9%)	3.3	(3.0, 3.6)		
Unknown	352	72 (20.5%)	n.i^§^			

**Notes:**

* OR, odds ratio.

† aOR, adjusted odds ratio.

‡ ref., referent category.

§ n.i, category not included in the analysis.

¶ Variable not included in the multivariable model due to collinearity.

### Spatial and spatial–temporal models

The spatial–temporal model that included time element (year of HIV diagnosis), spatial layer with contiguous districts and covariates produced the lowest DIC (305) compared to a spatial model without covariates (DIC 1319), a generalized linear model that had only the outcome with covariates (DIC 1153), spatial–nontemporal model with covariates (DIC 325), and spatial–temporal model (DIC 306) (Table 3).

**Table 3:** Model comparison using deviance information criterion (DIC) to identify best fitting model.

Model type	DIC	Effective parameters	Model choice
Model 1—A generalized linear model (nonspatial)	1,153	4.0	Fourth
Model 2—Spatial model without covariates	1,319	11.8	Fifth
Model 3—Spatial–temporal model without covariates	306	59.7	Second
Model 4—Spatial–nontemporal model with covariates	325	62.3	Third
Model 5—Spatial–temporal model with covariates	305	58.8	First*

**Note:**

* Best fitting model with lowest DIC (>10) from the next model of a different nature (model 4).

**Figure 3:**
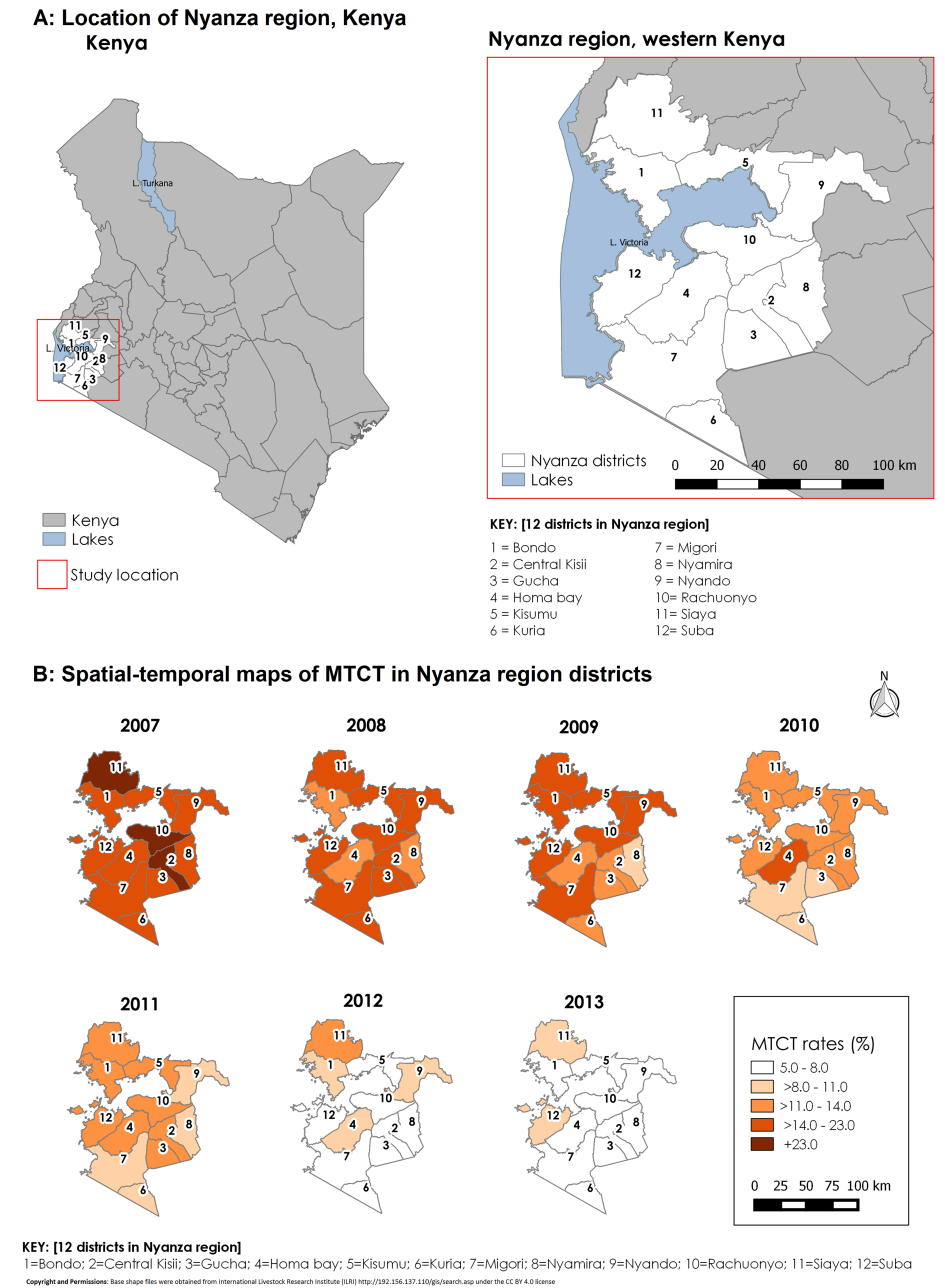
Study location and spatial–temporal trend of fitted MTCT rates in Western Kenya, 2007–2013. (A) Figure shows the study location in relation to the rest of Kenya. (B) Shows spatial–temporal trend of fitted MTCT rates.

Figure 3 contains choropleth maps for fitted MTCT rates for the seven-year period. Darker shades indicate higher HIV-infection rates. Infection rates gradually decreased from 2007 and by 2013, only two districts (Siaya and Suba) had rates higher than 8.0%.

### Comparison of raw and fitted MTCT rates

Spatial–temporal and covariate-adjusted MTCT rates showed a gradual reduction from 19.8% in 2007 to 7.2% in 2013 compared to nonadjusted rates which reduced from 19.7% in 2007 to 7.0% in the seven-year period (Table 4). The overall reduction in MTCT rates over time was by 63.6%. However, this average trend compares at aggregate level but not over space and time. Both unadjusted and adjusted revealed that the reduction was more evident in some districts than others. We demonstrated similar reduction in standardized MTCT rates by using the number of infected infants for each district out of estimated live births (Table 5).

**Table 4:** Comparison of raw and fitted MTCT by district and year among infants in western Kenya, 2007 and 2013.

District	Raw MTCT rates (%)				Adjusted MTCT rates (%)			
	2007	2013	Reduction	Rank*	2007	2013	Reduction	Rank*
Total	19.7	7.0	64.3%	–	19.7	7.2	63.6%	–
Bondo	21.3	6.7	68.5%	4	17.6	7.8	55.8%	10
Kisii	17.9	6.6	63.1%	7	23.5	6.3	73.2%	3
Gucha	17.6	6.8	61.4%	8	17.1	7.2	57.6%	8
Homa bay	18.0	8.3	53.9%	11	20.6	7.9	61.7%	6
Kisumu	19.1	6.8	64.4%	6	21.4	5.5	74.3%	1
Kuria	33.3	7.4	77.8%	1	15.1	5.4	64.4%	5
Migori	17.6	7.4	58.0%	9	22.1	5.8	73.6%	2
Nyamira	18.5	4.9	73.5%	2	17.3	7.1	58.9%	7
Nyando	22.6	7.9	65.0%	5	16.2	7.4	54.6%	11
Rachuonyo	19.7	8.3	57.9%	10	23.2	6.7	71.2%	4
Siaya	10.1	7.2	28.7%	12	23.7	10.9	54.1%	12
Suba	20.2	6.0	70.3%	3	19.2	8.3	57.0%	9

**Note:**

* Rank = highest to lowest MTCT reduction rates (2013 minus 2007 rate).

**Table 5:** Absolute transmissions and transmission rates per 100,000 live births by district among infants in western Kenya, 2013.

District	Estimated live births in 2013*	Women tested for HIV in 2013^†^	HIV+ women in 2013	Infants tested in 2013	Absolute transmission (number infected)	Transmission rates per 100,000 live births^‡^	Rank^§^
All	275,169	203,069	15,136	17,129	1,231	447	–
Bondo	13,262	9,925	1,372	1,739	116	875	11
Kisii	36,841	25,143	622	701	46	125	3
Gucha	17,231	17,316	375	293	20	116	2
Homa bay	43,423	13,159	1,257	1,968	163	375	5
Kisumu	24,931	29,599	2,882	2,469	167	670	9
Kuria	11,696	13,774	214	473	35	299	4
Migori	30,193	26,391	2,503	2,582	190	629	7
Nyamira	26,640	15,827	354	445	22	83	1
Nyando	19,063	10,307	1,208	1,286	102	535	6
Rachuonyo	17,243	12,658	1,451	1,457	121	702	10
Siaya	24,984	21,589	1,998	2,276	163	652	8
Suba	9,662	7,381	900	1,440	86	890	12

**Notes:**

* Kenya population estimates 2010–2018.

† PEPFAR annual progress report (APR 2013) data.

‡ ${\rm{Transmission}}\;{\rm{rate}}\;{\rm{per}}\;100,000\;{\rm{live}}\;{\rm{births}}\; = {{{\rm{Absolute}}\;{\rm{transmission}}\;{\rm{in}}\;2013} \over {{\rm{Estimated}}\;{\rm{live}}\;{\rm{births}}\;{\rm{in}}\;2013}} \times 100,000$.

§ Ranked from lowest to highest case rates.

### Standardized HIV MTCT rates per 100,000 live births

By 2013, the program had achieved an estimated 447 HIV standardized MTCT rates per 100,000 live births (Table 5). Nyamira, Gucha, and Kisii districts had the lowest HIV MTCT rates in 2013 while the highest MTCT rates were in Suba, Bondo, and Rachuonyo districts.

## Discussion

We identified geographical variations and a significant decline in MTCT rates in the seven-year period. The fastest progress occurred in more recent years from 2011 to 2013. We estimated a reduction by 51.0% in overall fitted MTCT rates between the years 2009 and 2013. This reduced transmission at the later period for our analysis is comparable to 55% reported in Kenya over the period 2009–2015 (Joint United Nations Programme on HIV/AIDS (UNAIDS), 2016a). While the overall MTCT rate up to infancy for the Nyanza region was ∼7% in 2013, our results show great progress towards e-MTCT. PMTCT through widespread use of ART can reduce the rate of vertical transmission to <5% in breastfeeding populations (World Health Organization (WHO), 2010). Our study showed increased use of ART for life which is one of the factors that could have led to reduced MTCT. However, using percentage rates may not appropriately measure the progress since it does not take into account the underlying population. We additionally used standardized MTCT rates per 100,000 live births. Out of 12 districts, none had attained e-MTCT impact target of ≤50 pediatric infections per 100,000 live births. By 2013, of the 12 districts, only Kuria, Kisumu, and Migori were close to attaining e-MTCT goal of <5% MTCT rate. Standardized MTCT rate was still high at 447 per 100,000 live births and above the target of 50 new infections per 100,000 live births. This rate is moderate and comparable to estimated 384 infants per 100,000 live births in South Africa (Goga et al., 2016). The differences at district level for fitted MTCT rates and standardized MTCT rates per 100,000 live births may due to the differences with which the methods are applied with the latter taking into account estimated live births.

Our challenge then is to understand the drivers of these varied results despite uniform policy and little variation in resource availability. In understanding disparities in PMTCT progress, ecological studies such as ours have previously been proposed (Hampanda, 2013). In our setting, for example, one such study has identified social barriers which may slow progress towards e-MTCT. These include individual level factors such as mothers’ competing priorities including work affecting service utilization and medication adherence; family-related factors such as lack of support by male spouses and partners; community-related such as fear and stigma; and institutional factors such as negative attitudes by health workers (Onono et al., 2015), and accessibility of facilities due to distance (Gourlay et al., 2013). These issues have been identified and described in other low-income settings (Turan & Nyblade, 2013). Challenges in implementation of Option B+ in western Kenya have been described despite successful implementation. These have to do with health system readiness, e.g., same-day initiation into treatment, staffing, training, and resource constraints; service-centered challenges such as scolding of nonadherent patients and inconvenient operation hours (Helova et al., 2017).

Higher infection rates among infants tested after eight weeks after birth indicate high postnatal transmission during the breastfeeding period. In the more recent years where use of lifelong ART during pregnancy and after birth (Option B+) is common, transmission rates are expected to be lower than in previous years. The estimates and projections package has estimated that generally 50% or more of transmission is expected to happen after six weeks of delivery in pre-Option B+ population for the Nyanza region. Early testing for HEI is recommended for timely intervention. According to the final stock-taking report on e-MTCT, by 2015, only four countries in East and Southern Africa were meeting targets of early testing to over 50% of HEI (Claessens et al., 2014). In our study, reduction in MTCT corresponded to a reduction in use of sdNVP use over time and adoption of more efficacious regimens. Our analyses covers a pre-Option B+ phase hence better progress would be expected during full implementation of Option B+. This progress towards use of efficacious regimens was in response to recommendations for use of universal ART (World Health Organization (WHO), 2010; DeCock et al., 2000). In this regard, Kenya has identified PMTCT goals for e-MTCT including implementing guidelines and improving EID and pediatric ART (Claessens et al., 2014).

Our best fitting model was a spatial–temporal model with covariates and had the least DIC (by over 10 points) from the next model of a different nature (spatial–nontemporal model). Therefore, this model was better in explaining geographical variation in MTCT rates over time. The fit observed for the spatial–temporal model with covariates can be explained by the way the PMTCT program has been implemented within the 12 districts. Initial PMTCT program implementation started in former southern Nyanza districts namely; Homa Bay, Suba, Migori, Gucha, Kisii, Nyamira, and Kuria districts. The spike in rates in Homa Bay district in 2010 may have been the result of intensified efforts in EID leading to diagnosis of more HIV-infected infants who may have been missed previously. After 2010, the trend shows a gradual reduction of MTCT rates for most districts up to the end of 2013. However, there was a spike for Gucha district in 2011 after a gradual decline up to 2010. By 2013 though, the decline to the 5–8% MTCT rate category was observed for most (10/12) districts. The highest rates by 2013 were in Siaya and Suba districts. Despite substantive program investments in these districts, the impact could be less due to rural nature of the district. Lower rates were observed in contiguous districts that were further away from Lake Victoria.

We acknowledge that our data have limitations. Routine program data may lack high-level quality due to missing values, although by focusing on a specific laboratory request form we had more complete results than routine patient records. In our data, we used the first infant PCR test and not the final one at 18 months. Our data does not describe final transmission rate, but lets us examine important factors in e-MTCT including early diagnosis. The variables included in the models are not exhaustive in explaining reducing MTCT rates including health seeking behaviors and other structural factors such as distance to health facility. We did not also include infant ART variable due to lack of sufficient data. However, we did include the variables that have been shown to be most important in impact on reducing MTCT rates in published literature such as level of facility (Lerebo et al., 2014), however, due to lack of data, we did not consider structural factors (Aarons et al., 2016), nor retention associated factors (Obai, Mubeezi & Makumbi, 2017) and other maternal related factors (Lerebo et al., 2014). We also acknowledge that DIC only measures the goodness of fit and cannot be used singly to conclusively indicate that the spatial–temporal model with covariates was best. We however tested for sensitivity with resulting similarity in final fitted rates.

## Conclusion

To the best of our knowledge, there is no comparable geospatial–temporal analysis for MTCT in SSA countries. We have revealed geographic disparities in progress attained in reductions of MTCT in this high-burden, low-resource setting. Rigorous country-wide analyses of this nature will be a useful addition to unveiling progress towards e-MTCT. Taking into account adoption and use of national PMTCT program guidelines, the spatial disparities revealed in our study imply the need to consider location-specific challenges.

## Supplemental Information

10.7717/peerj.4427/supp-1 Supplemental Information 1 Spatial-statistical analysis approach.

10.7717/peerj.4427/supp-2 Supplemental Information 2 Aggregate data for spatial model-fitting.

## Additional Information and Declarations

### Data Availability

The following information was supplied regarding data availability: Figshare: https://figshare.com/s/909012b948a0e9920955.
