# Supplementary material for: Spatial–temporal trend for mother-to-child transmission of HIV up to infancy and during pre-Option B+ in western Kenya, 2007–13
Source: PeerJ. 2018 Mar 13;6:e4427. doi: 10.7717/peerj.4427 (PMC5861528; doi:10.7717/peerj.4427)
Supplement: Supplemental Information 1 [file peerj-06-4427-s001.docx]

**Appendix I. Spatial-statistical analysis approach**

Let $Y_{it}$ denote the number infants infected with HIV in district $i$ and time $t$ out of $N_{it}$ children at risk,$i=1,\ldots,12$ and $t=1,\ldots,7$. We assumed that $Y_{it}$ has a Poisson distribution with a risk of infection $\theta_{it}$ . That is, $Y_{it}\sim Poisson\left( E_{it} exp( \theta_{it}) \right)$, where $E_{it}$ denotes the expected number of infants infected with HIV in district $i$ and time $t$. We model the risk of HIV infection using Hierarchical spatial Poisson regression models that accounts for excess heterogeneity and similarity over space and time. A class models were fitted to the data to assess the effects of selected covariates on the outcome of interest. These were based on a variant of the [Knorr-Held^[[1]](#footnote-2)^](#_ENREF_2)  formulation expressed as:

$$log\left( Y_{it} \right)=log\left( E_{it} \right)+\sum_{j=1}^{n_{f}} f^{\left( j \right)}\left( u_{ji} \right)+\sum_{k=1}^{n_{\beta}} {\beta_{k}z}_{ki}+\upsilon_{i}+\nu_{i}+{\gamma_{t}+\phi}_{t}$$

where $\left\{ f^{\left( j \right)}\left( . \right) \right\}$’s are unknown functions of the covariates $u$, the $\left\{ \beta_{k} \right\}$’s represent the linear effect of covariates $z,$ $\nu_{i}'$s are spatial unstructured components, which are independent and identically distributed with zero mean and unknown precision, $\tau_{\nu}$; and $\upsilon_{i}'$s is spatially structured component which is assumed to vary smoothly from region to region. To account for such smoothness $\upsilon_{i}'$s are modelled as an intrinsic Gaussian Markov random field with unknown precision, *τ* *_s_*. In this formulation, $\phi_{t}$ represents temporally unstructured components which are independent and identically distributed with zero mean and unknown precision, $\tau_{\phi}$; and $\gamma_{t}$ is the temporally structured effect, modelled dynamically using a random walk through the following structure:

$$\gamma_{t}|\boldsymbol{\gamma}_{t-1}\sim N\left( \gamma_{t+1},\tau_{\gamma} \right)\mathrm{for} t=1$$

$$\gamma_{t}|\boldsymbol{\gamma}_{t-1}\sim N\left( \frac{\gamma_{t-1}+\gamma_{t+1}}{2},\frac{\tau_{\gamma}}{2} \right)\mathrm{for} t=1$$

$$\gamma_{t}|\boldsymbol{\gamma}_{t-1}\sim N\left( \gamma_{t-1},\tau_{\gamma} \right)\mathrm{for} t=12$$

Estimation of parameters was carried out using the Integrated Nested Laplace approximation approach. The latent Gaussian field for the model was $\xi=\left\{ f^{(j)}\left( . \right),\beta_{k},\upsilon_{i},\nu_{i},{\gamma_{t},\phi}_{t} \right\}$ with hyperparameter vector $\theta=\left\{ \tau_{\beta},\tau_{\upsilon},\tau_{\nu},\tau_{\gamma},\tau_{\phi}, \right\}$. Vague independent Gamma priors are assigned to each of the elements in *ϑ*.

The model was also expanded to include an interaction between space and time as follows:

$$log\left( Y_{it} \right)=log\left( E_{it} \right)+\sum_{j=1}^{n_{f}} f^{\left( j \right)}\left( u_{ji} \right)+\sum_{k=1}^{n_{\beta}} {\beta_{k}z}_{ki}+\upsilon_{i}+\nu_{i}+\phi_{t}+\delta_{it},$$

Where $\delta_{it}\sim N\left( 0,\tau_{\boldsymbol{\delta}} \right).$

1. Knorr-Held, L., *Bayesian modelling of inseparable space-time variation in disease risk.* Statistics in medicine, 2000. **19**(17-18): p. 2555-2567 [↑](#footnote-ref-2)
